# The prognostic and clinical value of neutrophil-to-lymphocyte ratio (NLR) in ovarian cancer: A systematic review and meta-analysis

**DOI:** 10.5937/jomb0-46035

**Published:** 2024-06-15

**Authors:** Zihan Zhang, Jinghe Lang

**Affiliations:** 1 Chinese Academy of Medical Sciences, Union Medical College, Department of Obstetrics and Gynecology, Peking Union Medical College Hospital, Beijing, China

**Keywords:** neutrophil-to-lymphocyte ratio, ovarian cancer, prognosis, meta-analysis, systematic review, overall survival, progression-free survival, biomarker, odnos neutrofila i limfocita, rak jajnika, prognoza, metaanaliza, sistematski pregled, ukupno preživljavanje, preživljavanje bez progresije, biomarker

## Abstract

**Background:**

Ovarian cancer (OC) is a major gynecological malignancy with varying prognosis. The Neutrophil-toLymphocyte Ratio (NLR) has been proposed as a potential prognostic biomarker. This study aimed to evaluate the prognostic and clinical value of NLR in OC.

**Methods:**

A systematic review and meta-analysis were performed following PRISMA guidelines, including studies that evaluated the association between NLR and survival outcomes in OC patients. Search was performed in PubMed, Embase, Web of Science, and Cochrane Library databases. Quality assessment was done using Newcastle-Ottawa Scale (NOS). Heterogeneity was assessed, and pooled hazard ratios (HRs) were calculated using fixed or random-effects models as appropriate.

## Abstract (sr)

**Metode:**

Sistematski pregled i meta-analiza su obavljeni u skladu sa smernicama PRISMA, uključujući studije koje su procenile povezanost između NLR i ishoda preživljavanja kod pacijenata sa OC. Pretraga je obavljena u bazama podataka PubMed, Embase, Web of Science i Cochrane Library. Procena kvaliteta je urađena korišćenjem Njukasl-Otava skale (NOS). Heterogenost je procenjena, a udruženi odnosi opasnosti (HRs) su izračunati korišćenjem modela fiksnih ili slučajnih efekata prema potrebi.

**Rezultati:**

Analizirano je dvadeset studija koje su uključivale različite etničke pripadnosti, uzraste i veličinu uzorka. Utvrđeno je da je visok NLR u obrnutoj korelaciji sa ukupnim preživljavanjem (OS) (HR=1,21, 95% CI 1,09-1,34, P<0,001) i preživljavanjem bez progresije (PFS) (HR=1,20, 95% CI 1,03-1,38, P<0,001). Stratifikovane analize su pokazale jaču povezanost kod azijskih pacijenata, studije sa manjim veličinama uzoraka, mlađim pacijentima i višim graničnim vrednostima NLR.

**Zaključak:**

Meta-analiza sugeriše značajnu inverznu povezanost između NLR i ishoda preživljavanja kod pacijenata sa OC, naglašavajući potencijal NLR-a kao jednostavnog, isplativog prognostičkog biomarkera. Međutim, značajna heterogenost i uticaj zbunjujućih faktora naglašavaju potrebu za daljim istraživanjem.

## Introduction

Ovarian cancer (OC) is an insidious disease whose incidence has seen an alarming rise over the years, gradually but persistently evolving into a significant health concern. The most recent statistics of cancer trends in the United States reported that in 2020, an estimated 21,750 new cases of OC were identified, and tragically, about 13,940 deaths were attributed to this malignancy [1] [2]. This makes OC the fifth leading cause of cancer-related deaths among women and the principal cause of mortality in the context of reproductive system malignancies.

OC is notoriously difficult to detect in its early stages due to its covert nature, often presenting with non-specific symptoms or no symptoms at all until it has progressed substantially [3]. This fact, coupled with the lack of effective early screening methods, means that the majority of OC patients are diagnosed at advanced stages of the disease. As a consequence, the prognosis for these patients is typically poor, making the search for effective diagnostic and prognostic tools an area of pressing clinical concern. Inflammation has long been recognized as playing a critical role in the development and progression of many types of cancer [4]. This association between inflammation and malignancy has been the focus of scientific investigation since as far back as 1863, when it was proposed that malignant tumors could originate from sites of chronic inflammation. Over the years, substantial epidemiological evidence has accumulated, supporting this theory and suggesting that chronic inflammation is indeed intricately involved in the pathogenesis of various tumors [5].

As an emerging player in the understanding of inflammatory processes in malignancy, the Neutrophil-to-Lymphocyte Ratio (NLR) holds considerable potential as a systemic biomarker for inflammation in the context of cancer [6]. NLR is a readily available and cost-effective marker derived from routine complete blood counts, making it an attractive candidate for use in the clinic. Extensive research has indicated the significant prognostic value of NLR in a wide variety of cancers, including colorectal, prostate, breast, non-small cell lung cancer, as well as in solid tumors and advanced malignant tumors [6] [7]. The specific role of NLR in these cancers is considered to stem from its ability to mirror systemic inflammation and the state of the immune response. Systemic inflammation, as measured by the NLR, is increasingly recognized as an influential factor in the biology of various cancers. The inflammatory response, signaled by a high NLR, can contribute to an environment that is conducive to tumor growth, progression, and metastasis by promoting cellular proliferation, angiogenesis, and inhibiting programmed cell death or apoptosis [8]. These effects are largely mediated by pro-inflammatory cytokines and chemokines that are released by neutrophils and other cells of the immune system. Similarly, the immune response status is critical in the complex interplay of tumor development and progression. The lymphocyte component of the NLR provides an indication of the host's immune response, with lower lymphocyte counts potentially signifying a weakened immune response, thereby enabling tumor growth and spread. Hence, an elevated NLR, suggestive of a high neutrophil count and low lymphocyte count, could reflect an environment of sustained systemic inflammation and a suppressed immune response, creating a favorable setting for cancer progression. In the context of OC, the potential value of NLR as a prognostic tool is an area that is currently under extensive investigation. While some studies have pointed towards a possible role of NLR in predicting outcomes in OC, a consensus has yet to be reached [9]. Some studies suggest that a high NLR is associated with poorer survival rates in OC, but these studies have been limited by small sample sizes and heterogeneous patient populations, leading to inconsistent and potentially unreliable results.

While some studies have evaluated NLR's prognostic significance in OC, their findings have been limited by small sample sizes and have shown inconsistent results. This has led to a call for larger, more robust studies to more conclusively determine the role of NLR in OC prognosis. This research aims to not only provide more robust evidence regarding NLR's role in OC but also to explore its relationship with various clinical and pathological parameters. We hope that our findings will shed light on the potential application of NLR as a biomarker in OC, ultimately contributing to improved diagnosis, prognosis, and management of this devastating disease.

## Materials and methods

### Search strategy

Throughout the course of conducting our systematic review and the subsequent reportage of our findings, we strictly adhered to the guidelines delineated by the Preferred Reporting Items for Systematic Reviews and Meta-Analyses (PRISMA) [10]. We conducted an exhaustive search of four primary electronic databases, namely PubMed, Embase, Wxeb of Science, and the Cochrane Library, on January 6, 2023, without imposing any restrictions regarding the publication date. The search terms and syntax were individually customized to suit each specific database. For the PubMed database, we utilized the following search terms: (»Ovarian Cancer« OR »Ovarian Neoplasm« OR »Ovary Neoplasms«) AND (»Neutrophil-to-Lymphocyte Ratio« OR »NLR«). We placed no restrictions on language to ensure a comprehensive inclusion of relevant studies. Furthermore, we manually scrutinized the reference lists of pertinent articles to identify any additional studies that could be included in our analysis.

### Inclusion criteria

Studies included in the systematic review needed to meet the following criteria: 1) Population: Studies involving patients diagnosed with ovarian cancer, with no restrictions on age, ethnicity, or nationality; 2) Intervention: Studies evaluating the Neutrophil-to-Lymphocyte Ratio; 3) Outcomes: Studies providing data on overall survival (OS), progression-free survival (PFS), and clinical pathological parameters (such as tumor stage, grade, and pathological types).

The exclusion criteria were as follows: 1) Duplicate reports, studies of low quality, or those where data extraction is not possible; 2) Studies failing to provide survival data such as survival rates, survival curves, or hazard ratios (HR) with their 95% confidence intervals (95%CI); 3) Case reports, commentaries, expert opinion and narrative reviews.

### Data extraction

Two independent reviewers will undertake the screening of literature and extraction of data, conducting cross-verification for precision. Should any disagreements arise during this process, the reviewers involved will engage in discussions to reach a resolution, and, if necessary, may solicit the judgment of a third reviewer. The data to be extracted encompass: the authorship of the study, the publication year, the total number of cases investigated, the scrutinized outcome indicators, methodology employed in the study, the specific cut-off values utilized for the NLR, and the duration of follow-up reported in months. In instances where the published report does not contain the data of interest, the original study's investigators will be contacted via email to request the unpublished data. This approach is in line with the rigor and standards required in a meta-analysis study.

### Quality assessment

The quality assessment of the studies included in our meta-analysis will be meticulously conducted by two independent reviewers, employing the Newcastle-Ottawa Scale (NOS) as the evaluative tool [11]. The NOS, a widely recognized tool, assesses studies on nine factors distributed across three critical domains: selection, comparability, and outcome. These domains facilitate a comprehensive understanding of potential bias sources intrinsic to the studies. Upon this extensive evaluation, every study is allocated a quality score that varies between 0 and 9. The score interpretation is as such: studies with scores ranging from 0 to 3 are classified as low quality; scores between 4 and 6 reflect moderate quality; and high-quality research is indicated by scores from 7 to 9.

### Statistical analyses

The level of heterogeneity across studies was gauged using chi-square statistics and measured by the I2 value. If the I2 value was under 50% and the associated P-value was 0.10 or above, it signified the absence of substantial heterogeneity. In these instances, the fixed-effects model was utilized for the computation of the consolidated effect size. On the other hand, an I2 value of 50% or more, or a P-value less than 0.10, indicated significant heterogeneity. In such scenarios, we conducted either subgroup or sensitivity analyses to detect and resolve possible heterogeneity sources. When statistical heterogeneity was observed in isolation, the random-effects model was implemented to compute the consolidated effect size. The potential for publication bias was evaluated through assessing the symmetry of the funnel plot and Egger's test. All statistical analyses were two-sided, with a P-value less than 0.05 considered statistically significant. Data processing was performed using Stata version 17 (StataCorp, College Station, TX, USA).

## Results

### Search results and study selection

**Figure 1:**
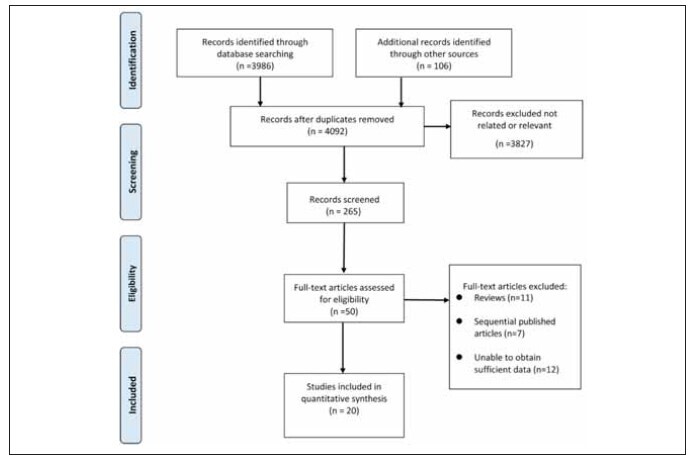
Selection process of included studies.

The preliminary search in the electronic databases resulted in the identification of 4092 potentially relevant articles. The elimination of duplicate articles, scrutiny of titles and abstracts, as well as rigorous application of the inclusion and exclusion criteria, whittled this down to 50 pertinent literatures. After a more thorough examination, a further 30 were deemed unsuitable. Consequently, 20 articles were selected for the final inclusion in the study [12] [13] [14] [15] [16] [17] [18] [19] [20] [21] [22] [23] [24] [25] [26] [27] [28] [29] [30] [31]. The process and outcomes of the literature screening are visually represented in Figure 1.

### Study characteristics

Table 1 presents the characteristics of studies included in the meta-analysis. These studies, published between 2009 and 2018, employed different methods to establish the cutoff value, most commonly using Receiver Operating Characteristic (ROC) curves. The cutoff values reported in these studies ranged from 2.3 to 5.25. The follow-up period varied widely across studies, with some studies not reporting this information. For the studies that did report the follow-up period, the range was wide, with the shortest being 0.1 month and the longest 192.9 months. Both OS and PFS were analyzed as outcomes in these studies, with HRs and 95% CIs reported. The OS HRs ranged from 0.29 to 14.13, while the PFS HRs ranged from 1.05 to 6.87. It is notable that some studies did not report either OS or PFS results.

**Table 1:** Characteristics of studies included in the meta-analysis. OS: Overall Survival; PFS: Progression-Free Survival; HR: Hazard Ratio; CI: Confidence Interval; ROC: Receiver Operating Characteristic; Quarta: Quartile method; »NR« indicates information was not provided.<br>Follow-up duration was reported as a range where available, otherwise the exact duration was used.

Author	Year	Method	Cutoff<br>Value	Follow-up Period<br>(months)	OS: HR (95% CI)	PFS: HR (95% CI)
KIM et al. [14]	2018	ROC	3.8	NR	1.89 (1.11~3.24)	1.25 (0.86~1.83)
ZHOU et al. [12]	2018	ROC	3.08	NR	1.38 (1.08~1.77)	1.38 (1.10~1.72)
KOMURA et al. [13]	2018	ROC	4	NR	-	1.58 (1.06~2.37)
LI et al. [15]	2017	ROC	5.25	49.5 (0.1~175.3)	1.39 (1.13~1.71)	-
BADORA-RYBICKA et al. [23]	2016	ROC	2.3	9.3	1.09 (0.98~1.22)	1.22 (1.08~1.39)
EO et al. [22]	2016	ROC	4.28	60	2.01 (1.18~3.43)	2.34 (1.45~3.77)
FENG et al. [21]	2016	ROC	3.24	29 (1~115)	1.19 (0.94~1.50)	1.25 (1.05~1.48)
MIAO et al. [19]	2016	ROC	3.02	72 (61~97)	1.62 (1.14~2.30)	1.73 (1.23~2.45)
PAIK et al. [17]	2016	ROC	3.91	52.5 (4~156)	1.07 (1.03~1.11)	1.05 (1.02~1.08)
WANG et al. [16]	2016	ROC	3.43	60	2.90 (1.66~5.05)	2.11 (1.29~3.46)
NAKAMURA et al. [18]	2016	ROC	3.91	NR	14.13 (1.21~165.40)	-
ZHANG et al. [24]	2015	ROC	3.4	43	2.17 (1.55~3.05)	-
KIM et al. [20]	2016	ROC	2.8	46 (6.1~192.9)	0.29 (0.13~0.68)	0.49 (0.25~0.99)
WANG et al. [25]	2015	Quarta	3.77	41.3 (3.3~70.4)	8.57 (2.81~26.14)	6.87 (2.64~17.91)
WILLIAMS et al. [26]	2014	ROC	3.6	6	1.37 (1.06~1.76)	-
KOKCU et al. [27]	2014	NR	2.7	NR	0.92 (0.69~1.24)	-
RAUNGKAEWMANEE et al. [28]	2012	ROC	2.6	14.7 (0.1~94.4)	1.17 (0.63~2.19)	1.12 (0.61~2.07)
ASHER et al. [30]	2011	Log-rank<br>test	4	24.5 (0.3~19.1)	0.87 (0.52~1.44)	-
THAVARAMARA et al. [29]	2011	ROC	2.6	50	0.70 (0.30~1.60)	0.70 (0.30~1.40)
CHO et al. [31]	2009	ROC	2.6	20.9	8.42 (1.09~64.84)	-

### Results of quality assessment

The methodological quality of each included study was evaluated utilizing the Newcastle-Ottawa Scale (NOS). Broadly, we observed that four studies attained a score of 7, six studies reached a score of 8, and ten studies achieved the highest score of 9. None of the studies implemented blinding procedures and there was no indication of allocation concealment. Any potential biases related to funding were not apparent across the studies. Additionally, there were no instances of incomplete outcome data, premature termination of the study, or imbalances at baseline. A summary of the risk of bias and the associated ratios can be found in Table 2.

**Table 2:** The quality assessment according to NOS of each cohort study. Note: NOS: Newcastle-Ottawa Scale

Study	Selection				Comparability	Outcome			Total<br>score
	Representativeness<br> of the <br>exposed cohort	Selection <br>of the non - <br>exposed cohort	Ascertainment <br>of exposure	Demonstration <br>that outcome	Comparability <br>of cohorts	Assessment <br>of outcome	Was follow- <br>up long <br>enough	Adequacy <br>of follow up <br>of cohorts	
KIM et al. [14]	★	★	★	★	★★	★	★	★	9
ZHOU et al. [12]		★	★	★	★★	★	★	★	8
KOMURA et al. [13]	★		★		★★	★	★	★	7
LI et al. [15]	★	★	★	★	★★	★		★	8
BADORA-RYBICKA et al. [23]	★	★	★	★	★★	★	★	★	9
EO et al. [22]	★	★	★	★	★	★	★	★	8
FENG et al. [21]	★	★	★	★	★★	★	★	★	9
MIAO et al. [19]	★	★	★	★	★★	★	★	★	9
PAIK et al. [17]	★	★	★	★	★★	★	★	★	9
WANG et al. [16]	★	★		★	★★	★		★	7
NAKAMURA et al. [18]		★	★	★	★★	★	★	★	8
ZHANG et al. [24]	★	★	★	★	★★	★	★	★	9
KIM et al. [20]	★	★	★	★	★★	★		★	8
WANG et al. [25]	★	★		★	★	★	★	★	7
WILLIAMS et al. [26]	★	★	★	★	★★	★	★	★	9
KOKCU et al. [27]	★	★	★	★	★★	★	★	★	9
RAUNGKAEWMANEE et al. [28]	★	★	★	★	★	★	★	★	8
ASHER et al. [30]	★	★	★	★	★★	★	★	★	9
THAVARAMARA et al. [29]	★		★	★	★	★	★	★	7
CHO et al. [31]	★	★	★	★	★★	★	★	★	9

### Association between NLR and Overall Survival

**Figure 2:**
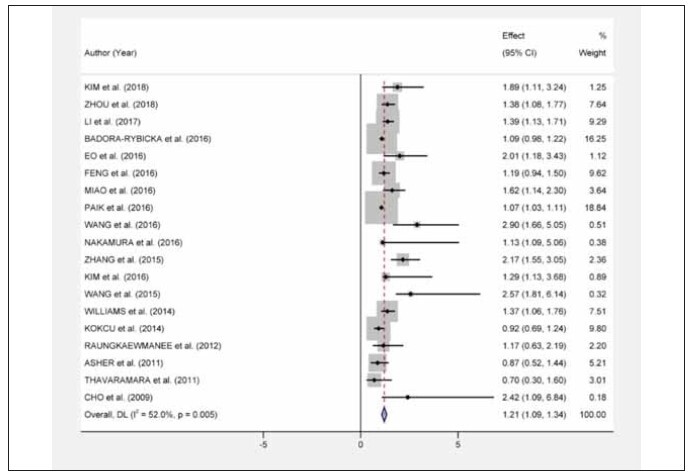
Forest plots of the association between NLR and Overall Survival.

In this study, we explored the relationship between the NLR and the OS in OC patients, across 19 distinct studies. The results highlighted an inverse correlation between high NLR and OS, indicating that OC patients with a low NLR have a longer OS than those with a high NLR (HR= 1.21, 95% CI 1.09–1.34, P<0.001, Figure 2). However, considering the substantial heterogeneity (I^2^=52.0%, P=0.005) among the studies, we further stratified the analysis based on various confounding factors including ethnicity, sample size, age, and the NLR cutoff value. While the association between NLR and OS was insignificant among Caucasian patients (HR=1.20, 95% CI 0.99–1.40, P=0.07, I^2^=57.8%), it was notably significant in Asian patients (HR=1.40, 95% CI 1.26–1.64, P=0.00, I^2^=80%). Studies with a smaller sample size (<200) showed a stronger association (HR=1.46, 95% CI 1.09–1.94, P<0.01, I^2^=81.8%) than those with larger sample sizes (≥200, HR=1.24, 95% CI 1.10–1.38, P<0.01, I^2^=65.5%). Moreover, both age groups (<55 and ≥55) demonstrated significant associations, but the link was more prominent in the younger cohort (HR=1.31, 95% CI 1.12–1.50, P<0.01, I^2^=81%). Lastly, a stronger association was observed in patients with an NLR value over 3 (HR=1.64, 95% CI 1.25–2.10, P<0.01, I^2^=83.3%) than those with an NLR value of 3 or less (HR=1.15, 95% CI 0.93–1.40, P=0.2, I^2^=67%) (Table 3).

**Table 3:** The results of subgroup analyses.

Subgroup	OS HR (95%CI)	P	I^2^(%)	P	PFS HR (95%CI)	P	I2(%)	P
Ethnicity								
Caucasian	1.20 <br>(0.99~1.40)	0.07	57.8	0.06	-	-	-	-
Asian	1.40 <br>(1.26~1.64)	<0.01	80	<0.01	1.39 <br>(1.18~1.58)	<0.01	81.8	<0.01
Sample Size								
200	1.24 <br>(1.10~1.38)	<0.01	65.5	<0.01	1.32 <br>(1.12~1.62)	<0.01	85	<0.01
<200	1.46 <br>(1.09~1.94)	<0.01	81.8	<0.01	1.34 <br>(1.00~1.72)	0.049	77.2	<0.01

55	1.26 <br>(1.00~1.54)	0.04	65	<0.01	1.39 <br>(1.10~1.70)	<0.01	50	0.16
<55	1.31 <br>(1.12~1.50)	<0.01	81	<0.01	1.25 <br>(1.04~1.42)	<0.01	84.8	<0.01
NLR Cutoff Value								
>3	1.64 <br>(1.25~2.10)	<0.01	83.3	<0.01	1.64 <br>(1.20~2.20)	<0.01	87	<0.01
≤3	1.15 <br>(0.93~1.40)	0.26	67	<0.01	1.20 <br>(0.94~1.55)	0.076	50	0.26

### Association between NLR and Progression-Free Survival

The association between NLR and PFS in ovarian cancer patients, presented in 13 different studies, is explored in this part of the meta-analysis. A significant conclusion from the pooled data indicates that OC patients with a low NLR have a longer PFS than those with a high NLR (HR=1.20, 95% CI 1.03–1.38, P<0.001). However, the presence of considerable heterogeneity (I^2^=71.9%, P<0.001) among the included studies necessitated further analysis through stratified subgroups. For the Ethnicity group, only data from the Asian cohort was available, which suggested an HR of 1.39 (95% CI: 1.18–1.58, P<0.01) indicating significantly shorter PFS for high NLR patients. Concerning sample size, studies with a size of ≥200 and <200 yielded HRs of 1.32 (95% CI: 1.12–1.62, P<0.01) and 1.34 (95% CI: 1.00–1.72, P=0.49), respectively. Age-wise, for patients ≥55 years, the HR was 1.39 (95% CI: 1.10–1.70, P<0.01) while for those <55 years, the HR was 1.25 (95% CI: 1.04–1.42, P<0.01). Regarding the NLR cutoff value, patients with NLR>3 had an HR of 1.64 (95% CI: 1.20–2.20, P<0.01), while those with NLR ≤3 had an HR of 1.20 (95% CI: 0.94–1.55, P=0.076) (Table 3).

### Sensitivity analysis

**Figure 3:**
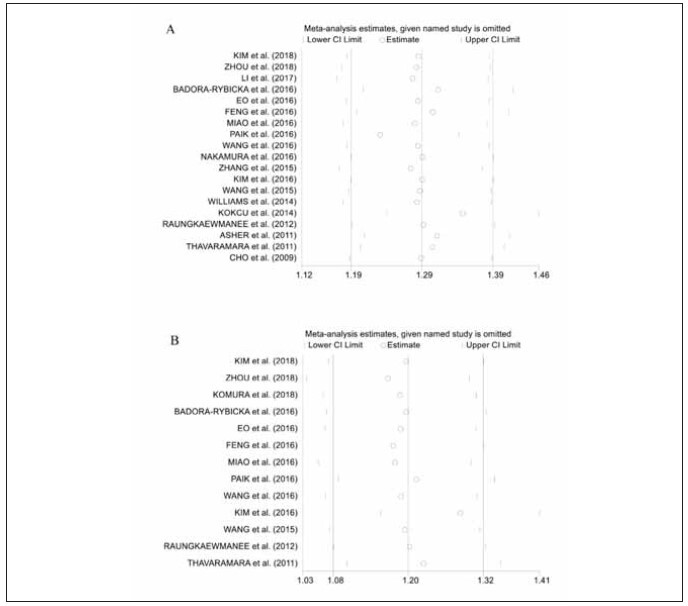
Sensitivity analysis of the association between NLR and Overall Survival (A) and Progression-Free Survival (B).

Due to the substantial heterogeneity detected amongst the studies incorporated in this meta-analysis, we employed a sensitivity analysis to evaluate the resilience and dependability of the compiled outcomes. In order to accomplish this, each study was sequentially omitted, with the cumulative effect measures recalculated for the remaining dataset. This stringent sensitivity analysis demonstrated that the collected outcomes were resilient and dependable, unaffected by the exclusion of any single study. This suggests that the overarching results were not unduly swayed by any single study, thereby amplifying the credibility of our conclusions. The persistent stability of the results throughout these analyses emphasizes the sturdiness of our primary outcomes and further consolidates the deductions made in this meta-analysis (Figure 3).

### Publication bias

**Figure 4:**
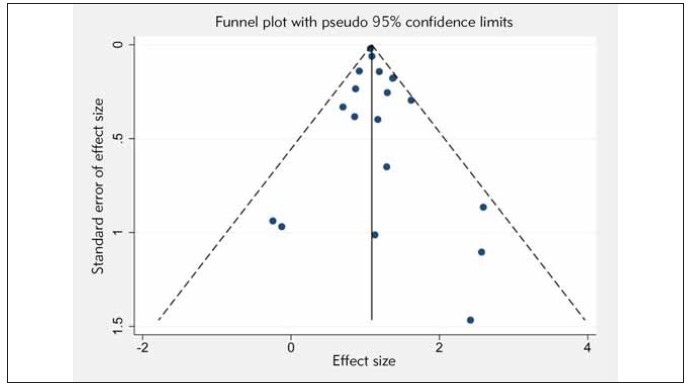
Funnel plot for publication bias in all included studies.

The symmetry of the funnel plots derived from the selected studies suggested the absence of noticeable publication bias (Figure 4). Furthermore, the application of the Egger’s linear regression test offered no evidence of significant publication bias in our meta-analyses under various parameters (P values for all exceeded 0.05). This adds further credence to the solidity and reliability of our meta-analysis outcomes.

## Discussion

The dynamic interplay between inflammation and cancer has long been recognized as a crucial driver in the etiology and progression of malignancies, including ovarian cancer [32]. This study aims to delve deeper into this relationship by examining the prognostic and clinical value of the NLR, a simple yet compelling systemic inflammation marker. Inflammatory mediators, such as cytokines and chemokines, are pivotal in nurturing an environment conducive to cancer cell proliferation. The uncontrolled multiplication of cancer cells, a hallmark of malignancies, is often facilitated by these growth factors, which are abundant in an inflammatory milieu [33]. Furthermore, these mediators activate oncogenic transcription factors like Nuclear Factor kappa B (NF-κB) and STAT3. Concurrently, oncogenes like Ras and Myc have been found to trigger inflammatory responses, reinforcing a vicious cycle of inflammation and cancer progression [34] [35].

Additionally, inflammation’s role in manipulating the immune response is also of note. Tumor-associated inflammation often suppresses anti-tumor immune responses, shifting the role of tumor-specific immune cells from a defensive stance to an offensive one, favoring tumor growth. Angiogenesis, another key contributor to tumor growth and metastasis, is also spurred by inflammation, ensuring an uninterrupted supply of nutrients to the burgeoning tumor mass [36]. The vital role of neutrophils and lymphocytes, the two key components of NLR, in this inflammatory-influenced cancer progression, cannot be understated. Neutrophils, by stimulating various inflammatory cytokines, curate an environment ripe for tumor growth. Meanwhile, lymphocytes are the vanguards of the body's immune response against the tumor, responsible for specific immune reactions aiming to obliterate the tumor. A shift in their balance, represented by increased NLR, often tips the scale towards an environment conducive to tumor growth, thus leading to a poor prognosis.

Our study highlighted a strong inverse association between NLR and survival outcomes in OC patients. We found that high NLR was significantly associated with reduced OS and PFS, implying a worse prognosis for OC patients with elevated NLR values. The biological rationale behind this relationship may lie in the complex interplay of inflammation and tumorigenesis, with the balance of neutrophils and lymphocytes reflecting the host's systemic inflammatory and immune status. Elevated NLR, representing either a heightened neutrophil-mediated inflammatory response or a diminished lymphocyte-mediated anti-tumor immune response, might foster a conducive environment for tumor growth and progression, resulting in poor survival outcomes. In our subgroup analysis, we observed a stronger association between NLR and survival outcomes in Asian patients, younger patients, smaller studies, and studies employing higher NLR cutoff values. It is noteworthy that ethnicity, age, study size, and NLR cutoff values could modulate the prognostic value of NLR. However, the underlying reasons for these differential impacts warrant further exploration. Moreover, our results underscore the importance of establishing a universally acceptable NLR cutoff value for predicting survival outcomes in OC. The wide range of cutoff values utilized across studies in our meta-analysis potentially contributes to the observed heterogeneity and might limit the generalizability of our findings. Establishing a consensus on the optimal NLR cutoff value will be crucial for the standardized use of NLR as a prognostic biomarker in clinical practice.

The heterogeneity among the included studies was another significant challenge. Our subgroup analysis, albeit helpful, could not fully elucidate the sources of this heterogeneity. Several potential factors were speculated to contribute, including geographic and ethnic variations, differences in the study populations and treatment regimens, variations in individual responses to treatment, and disparities in lifestyle factors. The use of different NLR cut-off values and follow- up periods across studies further compounded this heterogeneity. Our reliance on aggregated data for meta-analysis, instead of individual patient data, might have introduced an additional layer of error. We recommend future research to incorporate individual patient data whenever possible to minimize this potential source of error. More importantly, prospective cohort studies with uniform NLR cut-off values and follow-up periods are warranted to corroborate our findings.

The study focused on the prognostic value of NLR in ovarian cancer, but there is growing interest in its potential clinical utility. NLR has shown promise as a biomarker for predicting chemotherapy response, guiding surgical decision-making, and identifying patients who may benefit from immunotherapy. High pretreatment NLR is associated with poor response to chemotherapy and worse survival outcomes. Monitoring changes in NLR before and after treatment could provide important information on treatment efficacy. Incorporating NLR into clinical practice has the potential to improve patient outcomes by enabling personalized treatment decisions and optimizing treatment strategies. Determining a standardized cutoff value for neutrophil-to-lymphocyte ratio (NLR) in clinical practice is challenging due to the heterogeneity in patient populations, variations in laboratory methodologies, and different endpoints being assessed. The lack of consensus on the ideal threshold value makes it difficult to establish a universal cut-off for NLR interpretation in ovarian cancer patients. A personalized approach, considering individual patient characteristics and specific clinical goals, may be necessary when using NLR as a biomarker in ovarian cancer management. Future research should focus on large-scale studies with standardized methods to ensure consistency in NLR measurement.

Although we meticulously conducted our meta-analysis, we acknowledge several limitations. The inherent biases of retrospective studies, which constituted the majority of our included studies, cannot be ruled out. Furthermore, our reliance on aggregated data for analysis might have resulted in ecological fallacy. Also, we did not have access to individual patient data, precluding a patient-level meta-analysis which could have offered more in-depth insights. The availability of individual patient data for research purposes offers advantages such as comprehensive analysis, personalized medicine approaches, and improved understanding of disease progression. However, challenges around patient privacy and data harmonization must be addressed. Collaborative efforts promoting responsible data sharing can facilitate scientific advancements using individual patient data.

## Conclusions

In conclusion, our meta-analysis suggests a significant inverse association between NLR and survival outcomes in OC patients, underscoring NLR's potential as a simple, cost-effective prognostic biomarker. The association between NLR and ovarian cancer outcomes can be attributed to the inflammatory micro environment, compromised immune response, and tumor-immune interactions. NLR serves as a valuable prognostic marker for predicting ovarian cancer outcomes and guiding treatment decisions. Further research is needed to explore the therapeutic implications of targeting NLR in ovarian cancer management. However, the substantial heterogeneity across studies and the potential influence of various confounding factors warrant further exploration.

## Dodatak

### Conflict of interest statement

All the authors declare that they have no conflict of interest in this work.
